# Cytokines and Madness: A Unifying Hypothesis of Schizophrenia Involving Interleukin-22

**DOI:** 10.3390/ijms252212110

**Published:** 2024-11-11

**Authors:** Adonis Sfera, Kyle A. Thomas, Jacob Anton

**Affiliations:** Patton State Hospital, 3102 Highland Ave., Patton, CA 92369, USAjacob.anton@dsh.gov (J.A.)

**Keywords:** IL-22, microbial translocation, schizophrenia, hypothesis, gut permeability, aryl hydrocarbon receptor

## Abstract

Schizophrenia is a severe neuropsychiatric illness of uncertain etiopathogenesis in which antipsychotic drugs can attenuate the symptoms, but patients rarely return to the premorbid level of functioning. In fact, with each relapse, people living with schizophrenia progress toward disability and cognitive impairment. Moreover, our patients desire to live normal lives, to manage their daily affairs independently, date, get married, and raise and support a family. Those of us who work daily with schizophrenia patients know that these objectives are rarely met despite the novel and allegedly improved dopamine blockers. We hypothesize that poor outcomes in schizophrenia reflect the gray matter volume reduction, which continues despite antipsychotic treatment. We hypothesize further that increased gut barrier permeability, due to dysfunctional aryl hydrocarbon receptor (AhR), downregulates the gut barrier protectors, brain-derived neurotrophic factor (BDNF), and interleukin-22 (IL-22), facilitating microbial translocation into the systemic circulation, eventually reaching the brain. Recombinant human IL-22 could ameliorate the outcome of schizophrenia by limiting bacterial translocation and by initiating tissue repair. This short review examines the signal transducer and transcription-three (STAT3)/AhR axis and downregulation of IL-22 and BDNF with subsequent increase in gut barrier permeability. Based on the hypothesis presented here, we discuss alternative schizophrenia interventions, including AhR antagonists, mitochondrial transplant, membrane lipid replacement, and recombinant human IL-22.

## 1. Introduction

Most psychotropic drugs exert their action by attenuating the symptoms of severe mental illness (SMI) without influencing the longitudinal course of the disease. Acute psychotic symptoms are often resolved in a matter of days or weeks after the initiation of antipsychotic therapy, indicating that these agents are extremely efficacious for acute psychosis. For this reason, antipsychotics are likely to remain the golden standard in schizophrenia (SCZ) care for the foreseeable future, although the administration of these drugs may be different, as nanoparticles may utilized as delivery vehicles. However, the antipsychotic response in chronic mental illness is less robust, suggesting that despite treatment, the core pathology continues to progress unabatedly. Moreover, recovery is often understood differently by doctors and patients. While fewer delusions and less frequent auditory hallucinations are regarded as treatment successes by the former, patients hope for the restoration of functionality. For example, a recent study found that only 13.5% of schizophrenia (SCZ) patients achieve sustained recovery [1]. Along this line, a large meta-analysis of over on hundred and forty studies, looking at the entire 20th century, found that sustained recovery and stable employment have not improved with the advent of antipsychotic medication compared to the pre-psychotropic era, suggesting that symptomatic relief does not alter the longitudinal course of disease [2]. Since SCZ is a syndrome, individual differences exist among the patients in this spectrum. On the other hand, compared to mood disorders, the longitudinal progress of SCZ is less impressive [3]. 

Several phenomena characterizing schizophrenia (SCZ) are difficult to reconcile with the dopamine (DA) or serotonin (5-HT) model. These include premature cellular/neuronal senescence, gray matter volume (GMV) reduction, loss of mitochondria, disappearance of gamma band frequencies on electroencephalograms (EEGs), and decreased illness insight or anosognosia [4,5,6,7]. Although mostly measurable, these changes are infrequently discussed and hard to fit in the currently accepted paradigms. 

Sustained recovery, defined as return to the premorbid level of functioning without relapse, is a rare phenomenon in SCZ despite the availability of novel and more potent antipsychotic drugs. The continuous erosion of gray matter volume (GMV), unaffected by the available drugs, is likely the root cause of poor outcomes. For example, the prevalence of SCZ has increased by over 65% between 1990 and 2019, suggesting that the overall outcome is unaltered by the postsynaptic blockade of dopaminergic transmission [8].

Interestingly, ketamine, muscarinic agonists, and dopamine (DA) D2 receptor partial agonists, such as aripiprazole, have not been associated with GMV loss [9]. A new study reported that compared to electron acceptors, the antipsychotic drugs giving electrons away do not adversely affect the gray matter volume [10].

More than a century ago, Emil Kraepelin believed that SCZ was caused by toxins from the body compartments, such as the mouth, intestine, or genitals, that migrate to the brain, inducing pathology. In this regard, Kraepelin inadvertently predicted the microbiome and the role of microbial translocation in the pathogenesis of SCZ. However, the infectious model of this disorder drew limited attention until the launching of the Human Microbiome Project, the discovery of innate lymphoid cells (ILCs), and the sporadic psychotic episodes induced by the COVID-19 pandemic. Interestingly, Gram-negative bacteria or their components, such as lipopolysaccharide (LPS) and *E. coli* K99, were found in Alzheimer’s disease (AD) brains, indicating migration from the GI tract [11]. Moreover, altered cytokines, known for their major role in neuropathology, were found in the peripheral blood of SCZ patients, suggesting inflammatory pathology.

Interleukin-22 (IL-22) is a member of the interleukin-10 family, a cytokine produced by several lymphocyte types, including T helper (Th) 17 cells and innate lymphoid cells type 3 (ILC3). In the gut, IL-22 acts on intestinal epithelial cells (IECs), regulating mucus formation, barrier permeability, and synthesis of antimicrobial peptides, indicating that this cytokine functions as the master regulator of intestinal integrity [12]. 

The crosstalk between IL-22 and its receptor (IL-22R), a dimeric protein composed of IL-22R1 and IL-10R2, activates the JAK/STAT pathway, a critical antibacterial and antiviral system [13,14]. As IL22R contains IL-10R2, it can be cross-activated by IL-10, a cytokine previously connected to SCZ [9,10,11]. In addition, several studies have shown that IL-22 possesses neuroprotective properties, as it deactivates microglia, cells previously associated with psychosis [15,16,17].

IL-22, “the guardian of the gut barrier”, is abundantly expressed in the GI tract and the central nervous system (CNS). At the level of the BBB, IL-22 augments tight junctions (TJs), the molecular Velcro that keeps endothelial cells together to establish the barrier function. In addition, IL-22 ignites controlled inflammation as it participates in tissue repair, such as wound healing, which requires an inflammatory process. Moreover, long-term memory formation also needs inflammation, along with intact DNA repair mechanisms [18,19].

IL-22 enhances learning and long-term potentiation via phosphorylation of signal transducer and transcription-three (STAT3). This transcription factor connects IL-22 and aryl hydrocarbon receptor (AhR) to the brain-derived neurotrophic factor (BDNF). BDNF is a positive regulator of neurite growth, plasticity, and memory, justifying the title “brain fertilizer”, coined by psychopharmacologists. However, BDNF also has a dark side: it is part of the senescence-associated secretory phenotype (SASP), a proinflammatory secretome released by the aging cells. As such, BDNF can disseminate senescence throughout the brain parenchyma in a neurocrine/paracrine fashion. IL-22 can also induce cellular senescence to protect against cancer and/or fibrosis [20].

Aging cerebral endothelial cells (CECs) generate 50 times more BDNF than neurons and secrete this neurotrophin and SASP directly into the systemic circulation, spreading senescence throughout the body. 

Recent studies have linked premature cellular senescence to SCZ and schizophrenia-like disorders (SLDs), conditions marked by premature aging [18,19,20]. Senescent neuronal cells release SASP, which probably accounts for the low-grade inflammation found in SCZ.

This short review discusses IL-22 and BDNF in SCZ. Furthermore, based on the hypothesis described here, we propose alternative interventions for this disorder, including muscarinic agonists, mitochondrial transplant, membrane lipid replacement, and recombinant human IL-22.

## 2. Outcomes in Schizophrenia

Kraepelin called SCZ dementia praecox and believed that it was a neurodegenerative disorder with a poor prognosis and unimpressive functional recovery.

Resolution of symptoms and partial recovery, defined as six months of minimal symptomatology without a return to the premorbid level of functioning, is not identical to sustained recovery and is more or less attainable [21]. However, living independently, maintaining stable employment, going to school, working, getting married, and raising a family are goals rarely accomplished by patients with chronic SCZ [22,23,24]. For example, 33% of SCZ patients relapse during the first 12 months after an initial psychotic episode, 26% remain homeless at two-year follow-up, while five years after the first psychotic outbreak, only 10% are employed [25,26,27]. In addition, the continued existence of psychiatric state hospitals, almost a century after similar public institutions for infectious diseases have been closed, is proof of the concept that sustained recovery in SCZ is an exception rather than the rule [28,29].

SCZ is a neurodevelopmental disease that likely originates in utero and involves genetics, interacting with environmental factors (such as pathogens and toxins). For example, the offspring of women pregnant during the 1964 rubella epidemic in the US developed autism or SCZ at a rate higher than the general population [28]. Other viruses associated with SCZ include herpes simplex viruses (HSV) 1 and 2, cytomegalovirus (CMV), Epstein–Barr virus (EBV), human herpes virus 6 (HHV-6), and varicella zoster virus (VZV) [29,30]. Likewise, prenatal exposure to pollutants, such as plasticizers, may lead to CNS pathology, including SCZ [31,32]. Furthermore, the discovery of a virome in Broadman area 46 of patients with SCZ and dysbiosis further substantiates the links between neuropathology and pathogens [33].

**Figure 1:**
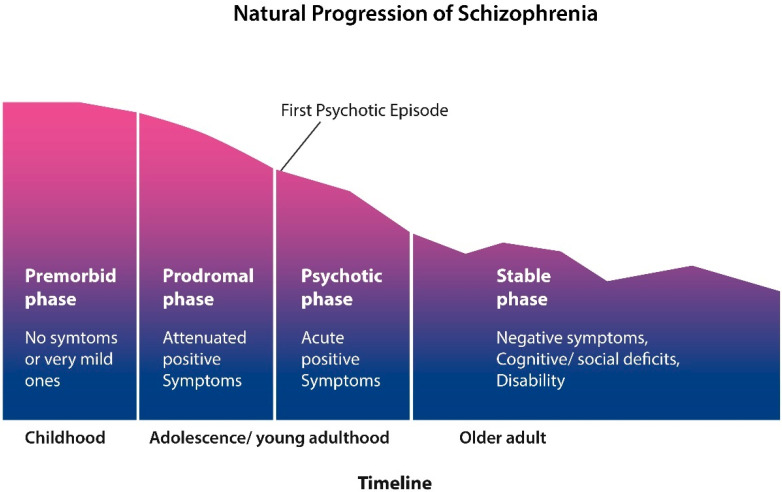
SCZ starts in early childhood with a premorbid phase with no symptoms or mild ones. Attenuated symptoms, such as social isolation, anxiety, and insomnia, mark the prodromal phase. This phase blends gradually into psychosis, during which patients are usually hospitalized many times for exhibiting positive symptoms. Around midlife, the positive symptoms gradually subside and are replaced by negative and cognitive manifestations (figure adapted from Liberman).

Clinically, the natural progression of SCZ evolves in four stages: an asymptomatic phase followed by a prodrome with mild but not overly psychotic symptoms, such as insomnia, peculiar beliefs, and internal preoccupation. Phase III is manifested as frank psychosis and comprises the first psychotic episode and subsequent relapses. Stage IV is marked by cognitive or negative symptoms and disability resembling a dementing illness (Figure 1).

## 3. The AhR/STAT3 Axis

Lack of insight into one’s illness or anosognosia is believed to reflect dysfunctional interoception, which is the awareness of the body’s internal state, including heart rate, respiration, and discomfort [34]. Interoception is opposed by exteroception, which is attention to the environment, mediated by senses [35]. Together, exteroception and interoception are driven by the salience network (SN) of the brain, a neuronal assembly anchored in the insular cortex (IC), anterior cingulate cortex (ACC), and some subcortical nodes that process affect and reward [36].

At the molecular level, interoception is mediated by sensors located at the level of biological barriers, including the gut and blood-brain barrier (BBB), which receive input from both outside and inside the body. One of such sensors is AhR, initially known as the dioxin receptor. However, it was lately identified as a transcription factor with many neuropathology-associated ligands, including neurotransmitters, psychotropic drugs, microbial metabolites, pollutants, plasticizers, indole, and vitamin D [37].

During the COVID-19 pandemic, the interest in interoception was reignited as patients with virus-induced cognitive impairment were often unaware of their deficits, suggesting that the pathogen alters the insight circuitry [38]. In addition, the study of frontotemporal dementia behavioral variant (bvFTD), in which von Economo neurons (VENs) are preferentially targeted, is characterized by impaired insight and gradual loss of emotional intelligence [39]. As VENs are located in the IC and ACC and participate in insight, this finding validates the earlier SCZ studies connecting IC and ACC with insight [40].

**Figure 2:**
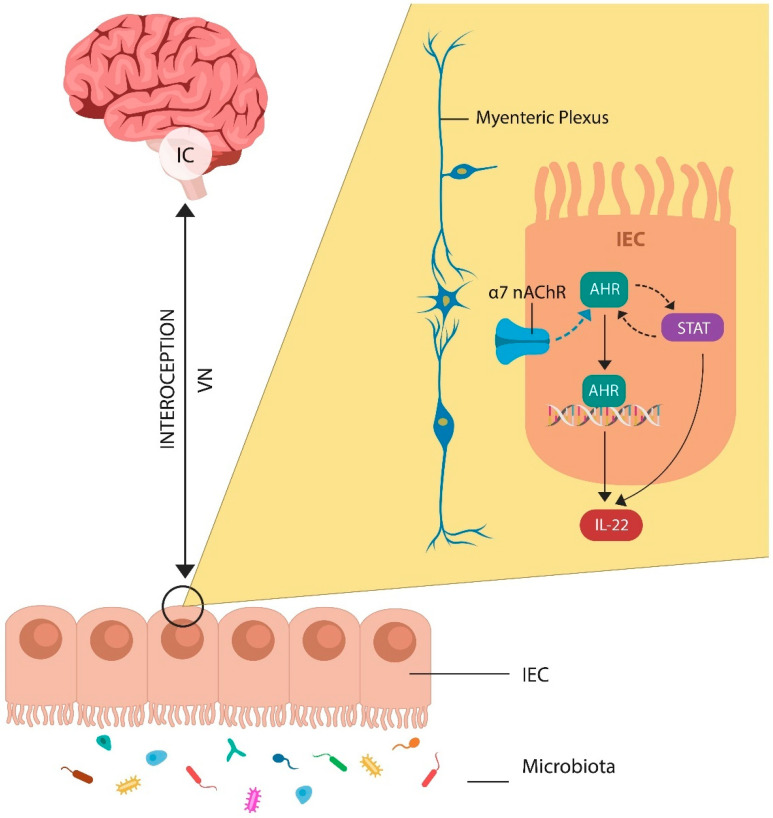
AhR is activated via the alpha-7 nicotinic acetylcholine receptor (α7 nAChR) expressed on intestinal epithelial cells (IECs). Vagal input into the gut barrier is mediated by the myenteric plexus, a gut–brain axis component linking the insular cortex (IC) to IECs. This link may enable GI tract interoception. AhR facilitates IL-22 transcription, while IL-22 regulates the gut barrier and the microbiota.

AhR is a transcription factor located in the cytosol of most cells, including intestinal epithelial cells (IECs). AhR is stabilized by two molecules of heat shock protein 90 (HSP90), which block its entry into the nucleus. When activated by ligands, AhR detaches from its chaperones and enters the nucleus, binding to the DNA to promote the transcription of genes, including IL-22. AhR and STAT3 activate each other, facilitating cholinergic transmission (Figure 2). In the GI tract, the action of the Vagus nerve is mediated by alpha-7 nicotinic acetylcholine receptors (α7 nAChRs) located in the myenteric plexus, a neuronal hub, often called the “gut brain” (Figure 2). Moreover, a novel study found that IC maintains a record of gut inflammations, suggesting that this area regulates abdominal interoception [41]. In this regard, IC plays a crucial role by mediating insight in IBD patients, further highlighting the link between this network and awareness [41,42]. 

IL-22 phosphorylates STAT3, which in turn phosphorylates BDNF, activating this neurotrophin. STAT3-activated BDNF is anti-inflammatory and promotes synaptic plasticity. In contrast, BDNF found in the composition of senescence-associated secretory phenotype (SASP) is neurotoxic [43].

## 4. Recombinant Human IL-22 for Schizophrenia

The realization that IL-22 regulates the permeability of the gut barrier, opposing microbial translocation, occurred during the 1980s HIV epidemic. HIV is characterized by the massive migration of gut microbes into the systemic circulation. It has been established that HIV induces apoptosis of ILC3, thus depleting IL-22. This, in turn, alters mucus production, inducing IEC senescence to defend against cancer [44].

Whether IL-22 is pro- or anti-inflammatory is currently unclear, as there have been contradictory study results. We reconcile these findings by showing that tissue and DNA repair requires IL-22-mediated inflammatory responses. In contrast, an anti-inflammatory microenvironment is needed to protect the tissue after healing. This is likely why IL-22 can generate “good inflammation”, which is both adaptive and short-lasting, as well as combat inflammatory responses. Indeed, recent studies have connected learning and memory to inflammation [45]. The relationship between IL-22 and BDNF is insufficiently characterized; however, it is known that IL-22 phosphorylates STAT3 (pSTAT3), a transcription factor necessary for memory formation. The pSTAT3 may contribute to interoception, i.e., awareness of body functions driven by the cholinergic system, and oppose GMV loss.

Our previous work hypothesized that SCZ might be initiated by aberrant AhR activation by endogenous or exogenous ligands, including intestinal or environmental toxins, such as LPS or plasticizers, respectively [46]. The following findings support this hypothesis:

1. SCZ is often comorbid with inflammatory bowel disease (IBD), conditions associated with increased gut barrier permeability and microbial translocation from the GI tract into host tissues, including the brain.
2. Translocation markers, including soluble CD14 (sCD14) and lipopolysaccharide-binding protein (LBP), are elevated in SCZ, indicating bacterial translocation.
3. Increased BBB permeability, documented in SCZ, enables translocated gut microbes to reach the brain.

**Figure 3:**
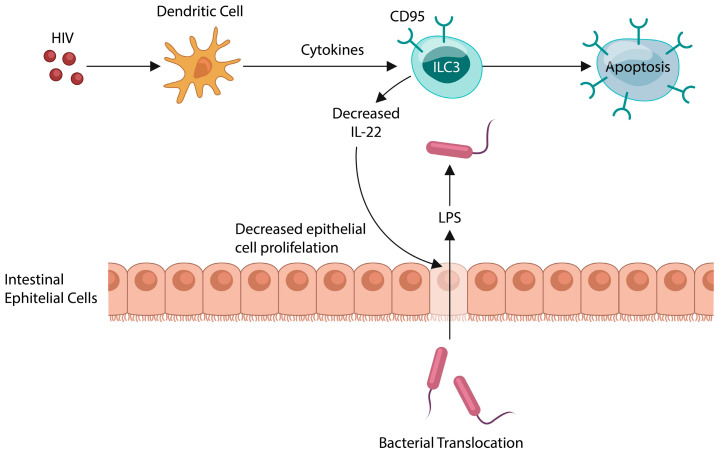
HIV induces the apoptotic loss of innate lymphoid cells, type 3 (ILC3), lowering IL22, the guardian of the gut barrier. This, in turn, promotes microbial translocation into the systemic circulation. Activated host immunity maintains a state of low-grade inflammation, a pathology documented in SCZ.

Along this line, the 2011 *Escherichia coli* (*E. coli*) outbreak in Germany has been associated with cases of new-onset psychosis, involving this microorganism in neuropathology. Moreover, new-onset or exacerbation of existing psychosis also occur in *E. coli*-associated urinary tract infections (UTIs), further linking this microbe to mental illness. On the other hand, IL-22 has been successfully used to restore the integrity of the gut barrier in various conditions, including IBD, HIV, and liver disease [47,48]. We construe that recombinant IL-22 would be effective for SCZ, as it limits the translocation of bacteria and their molecules as well as the BBB permeability (Figure 3).

Recombinant IL-22 is composed of two IL-22 molecules connected by a fusion protein; this conformation exerts better efficacy with reduced systemic side effects than individual IL-22 molecules [49].

## 5. BDNF and the Gut–Brain Axis

BDNF was discovered in 1982, and its role in CNS myelination was documented in 1997. It took another decade to realize that BDNF transduced the effects of antidepressant drugs, including ketamine, thus preventing hippocampal volume reduction [50]. Recently, a BDNF-antisense (BDNF-AS) mRNA was isolated, a molecule with actions opposed to BDNF with currently unknown functions [51]. We believe that BDNF-AS mediates inflammation via SASP-associated BDNF, the toxic secretome released by the senescent cells.

Abundantly expressed throughout the human GI tract, BDNF regulates gut motility and mucosal permeability [52]. Increased intestinal barrier permeability, documented in SCZ and IBD patients, is believed to reflect defective TJ molecules, including zonula occludens (ZO) and claudin-5, further linking barrier pathology to SCZ [53,54].

In the colon, BDNF preserves mucosal integrity and plays a crucial role in the pain associated with irritable bowel syndrome (IBS) [55]. IECs and goblet cells are the major BDNF producers in the GI tract, although smaller amounts are also released by the enteroendocrine cells.

In major depressive disorder (MDD), BDNF may work by limiting the translocation of bacteria and their components, such as LPS associated with depression by previous studies.

BDNF facilitates cholesterol efflux from astrocytes and ApoE expression but downregulates the cholesterol uptake in neurons, altering the lipidome of these cells [56]. As under physiological circumstances, cholesterol lowers aggressive behaviors; especially in psychiatric patients, low cholesterol and statins may contribute to iatrogenic aggression. The same can be said about SASP-derived BDNF. For example, cerebral endothelial cell (CEC)-derived BDNF was demonstrated to augment the exit of cholesterol from astrocytes, decreasing neuronal intake. SCZ has been associated with premature cellular senescence and lower CNS cholesterol, implicating BDNF in this pathology. Therefore, avoiding BBB-crossing statins in patients with SCZ and treating peripheral hypercholesterolemia with plasmalogens is something psychiatrists should consider. For example, ethanolamine plasmalogens exert cholesterol-lowering properties and prevent lipid peroxidation in plasma membranes, averting premature cellular senescence. Moreover, plasmalogens upregulate the “good” BDNF in the hippocampus, augmenting long-term potentiation and learning. 

Low BDNF activates STAT3, downregulating anti-inflammatory cytokines IL-10 and TNF beta, while upregulating the proinflammatory ones, IL-1 beta and TNF alpha. 

**Figure 4:**
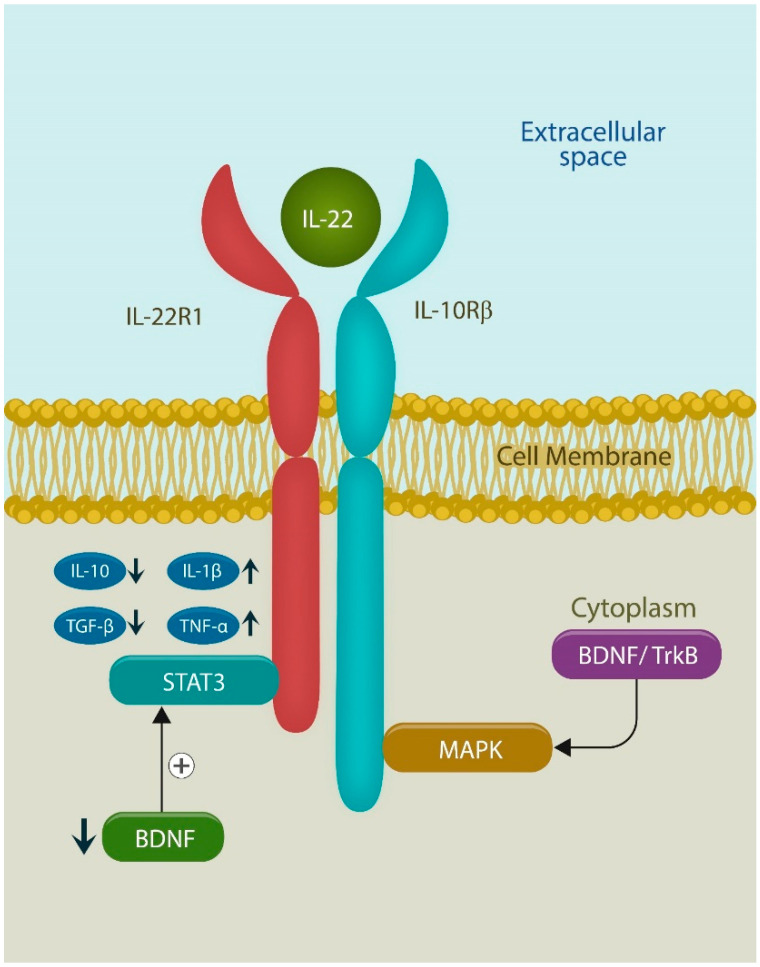
IL-22 receptor binds both IL-22 and IL-10. When IL-22 binds the receptor, IL-10 and TGF beta are reduced, while IL-1 beta and TNF alpha are upregulated, generating sufficient inflammation for wound healing and memory. IL-22-induced inflammation is probably caused by decreased BDNF and STAT3 activation. IL-10 receptor beta activates mitogen-activated protein kinases (MAPKs) via BDNF signaling with tropomyosin receptor kinase B (TrkB), its established receptor.

ILC3 releases IL-22 and IL-18. The IL-22 receptor attaches to both IL-10 and IL-22 (Figure 4). Interestingly, *Indigo naturalis* activates the IL-22 pathway, improving the symptoms of IBD.

## 6. Does Interleukin-22 Avert Brain Volume Reduction in Schizophrenia?

IL-22 and its receptor, present in various brain locations, play a crucial role in adult neurogenesis and the formation of new neurons in select CNS areas [57]. This action of IL-22 likely counteracts SCZ-associated GMV reduction, making it a promising intervention. Furthermore, IL-22’s ability to phosphorylate STAT3 (pSTAT3) and indirectly increase BDNF suggests a role in preventing brain parenchymal atrophy. Given that BDNF levels are reduced in both medicated and unmedicated SCZ patients, IL-22 could potentially prevent neuronal death by boosting BDNF levels, offering a major advantage in SCZ treatment [58]. 

IL-22-mediated STAT3 phosphorylation in IECs links the AhR/STAT3 axis to the neuroprotective cholinergic input. Interestingly, the new antipsychotic drugs, muscarinic agonists, such as the recently FDA-approved Cobenfy, are likely to avert the GMV reduction induced by DA blockers, indicating that IL-22 averts microbial translocation by more than one mechanism [59]. 

The microbial translocation hypothesis of SCZ can explain some SCZ characteristics that are difficult to reconcile with the DA model. These include the higher prevalence in urban than rural areas, the association with viral and microbial illness and toxicants, and high comorbidity with IBD. Recombinant IL-22 may improve SCZ outcomes, as it will likely address the pathogenetic factors rather than symptomatology.

## 7. Interventions

Treatment with cytokines and cytokine-targeting therapies are currently utilized in chronic inflammatory diseases, many of which are comorbid with SCZ [60,61].

This section discusses several potential nondopaminergic therapeutic strategies, including membrane lipid replacement (MLR), mitochondrial transplant/transfer, natural and synthetic AhR antagonists, and recombinant human IL-22. 

## 8. Mitochondrial Transfer or Transplantation

**Figure 5:**
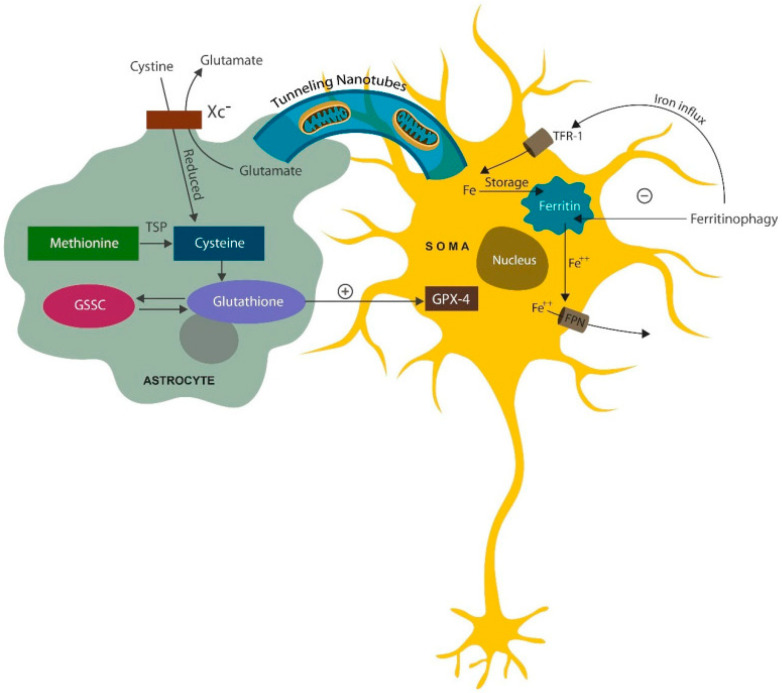
Astrocytes support neurons in many ways, including supplying healthy mitochondria via tunneling nanotubules., a process enhanced by SSRIs. Another support modality consists of donating antioxidants via the cysteine–glutathione pathway. Cystine enters the cell via the cystine/glutamate antiporter (Xc^−^) but can also be derived from methionine. Transfer of mitochondria and antioxidants to neurons helps avert cell death by ferroptosis (scheduled cell death due to excessive iron and lipid peroxidation by excessive iron).

The paucity of mitochondria in pyramidal neurons and astrocytes, especially in the anterior cingulate cortex (ACC), has been documented by SCZ postmortem studies [62,63]. Other research has shown that mitochondria can exit the cells and function in the extracellular compartment, playing a crucial role in immunity [64]. In addition, mitochondria can be transferred from astrocytes to neurons via tunneling nanotubules and extracellular vesicles (EVs), highlighting an underappreciated support mechanism for neuronal cells [65] (Figure 5). Aside from mitochondria, astrocytes transfer antioxidants to neurons, averting lipid peroxidation by the upregulated iron in aging neurons.

Mitochondrial transplantation studies started in the 1980s and focused on internalizing organelles from the extracellular environment [66]. During this time, HeLa cells were used as a source of mitochondria for cardiomyocytes. Mitochondrial DNA (mtDNA) serves as a marker of successful mitotransplantation. Mitochondrial transplantation has been attempted in animal models of SCZ but not in humans [67,68].

After traumatic brain injuries (TBIs), accelerated trafficking of mitochondria from astrocytes to neurons has been documented, emphasizing the supporting role of these cells. Interestingly, selective serotonin reuptake inhibitors (SSRIs) were demonstrated to facilitate mitochondrial transfer, highlighting a new antidepressant mechanism of action [69,70].

## 9. Membrane Lipid Replacement (MLR) with Plasmalogen

MLR refers to substituting oxidized lipids in the cell membrane bilayer with natural exogenous glycerophospholipids, often with plasmalogens and antioxidants.

Plasmalogens are more susceptible to oxidation than other phospholipids. They can precipitate a cascade of reactive oxygen species (ROS) that may contribute to further peroxidation of different lipids, leading to cell death.

Oxidized plasmalogen was found to lower cholesterol content in plasma and mitochondrial membranes, which, in patients with SCZ, could promote aggressive behaviors [71,72]. As mentioned above, decreased cholesterol was demonstrated to trigger aggression and violence in many patients with SCZ. Indeed, several studies have linked violence to low cholesterol, indicating that lipophilic (BBB-crossing) statins, including atorvastatin, could promote dangerous behavior in psychiatric patients [73,74,75,76,77]. Later, industry-sponsored studies found that statins were neuroprotective, antidepressant, and not related to aggression, hoping to counteract the previous findings. However, very few of these studies involved patients with severe mental illness taking lipophilic statins. More studies are needed to elucidate the statin–aggression link. It may be that oxidized but not healthy cholesterol triggers aggressive behavior via AhR activation, explaining the efficacy of MLR and plasmalogens in SCZ patients.

There was also discussion about statins causing depression, given that this condition increased by more than 300% after 1987, when the first commercial statin, lovastatin, received FDA approval [78]. Interestingly, Prozac, the first SSRI, was also released in 1987, and since 10% of the US population takes both statins and antidepressants, statins can likely alter the mood [79].

Lipid peroxides were demonstrated to change the biophysical properties of cell membranes. In contrast, MLR reverses oxidation-mediated biophysical changes in the plasma and mitochondrial membrane, restoring membrane composition and fluidity. Interestingly, some antipsychotic drugs, including phenothiazines, enter the lipid bilayer, correcting oxide-induced membrane curvatures and pores and preventing cell death.

Novel phenothiazines with antioxidant properties have been developed for cancer and may also benefit patients with SCZ [80]. These compounds could be incorporated into cell membranes along with other natural agents. For example (Table 1), in another study, we recommended Berberine and Kaempferol along with MLR, as this combination could benefit patients with SCZ by inhibiting glycogen synthase kinase-3 beta (GSK-3β) [81]. Moreover, the fluid-mosaic model of cell membranes explains how the natural glycerophospholipids intercalate globular proteins into the lipid bilayer, independently of proteins [82,83].

## 10. AhR Antagonists

**Figure 6:**
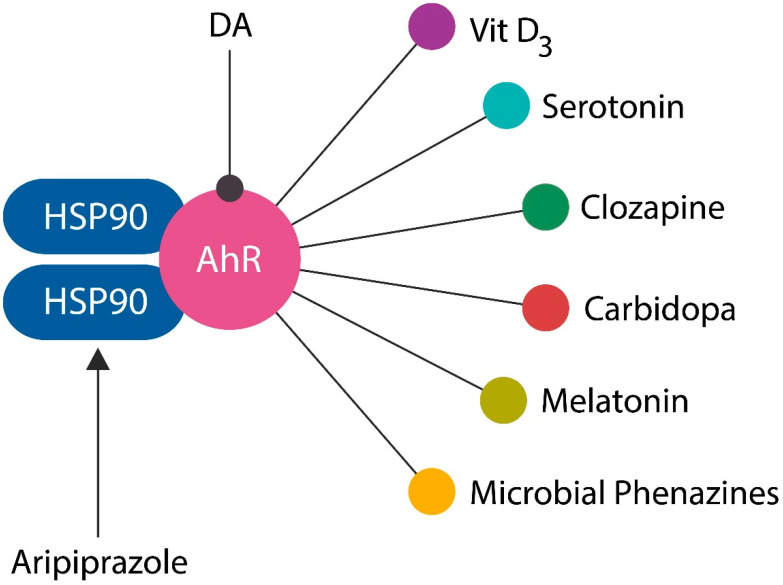
Numerous exogenous and endogenous ligands, including DA, vitamin D3, 5-HT, melatonin, clozapine, carbidopa, phenazines, and phenothiazines, activate AhR. Aripiprazole, on the other hand, binds heat shock protein 90 (HSP 90), an AhR chaperone.

AhR involvement in the pathogenesis of SCZ may lead to a unifying hypothesis that could explain the aspects of this disorder that are difficult to account for by the DA or serotonin (5-HT) model. For example, SCZ has a higher prevalence in urban than the rural areas, is associated with plasticizers and pollutants, is comorbid with IBD, exhibits a paucity of mitochondria, loses the gamma band on EEGs, and is more prevalent in colder areas of the world compared to the equator (Figure 6).

## 11. The Inbuilt Antipsychotic System

Phenazines are products of the commensal intestinal flora that are very similar to phenothiazines, the synthetic antipsychotic drugs. This suggests that gut microbes may engender an inbuilt antipsychotic system akin to endogenous opioids. 

Recently developed antioxidant phenothiazines are intended for cancer, cardiovascular disease, and antibiotic-resistant microorganisms, but they are likely beneficial for SCZ [80]. For example, propenyl-phenothiazine is a potent antioxidant with electron-donor capabilities that likely prevents GMV reduction in patients with SCZ. Moreover, a new category of tetracyclic and pentacyclic phenothiazines with antioxidant properties has been developed, suggesting their likely efficacy for cognitive and negative SCZ symptoms. For example, N10-carbonyl-substituted phenothiazines inhibit lipid peroxidation, suggesting enhanced antipsychotic efficacy [89].

## 12. Recombinant Human IL-22

Recombinant human IL-22 is currently in Phase II clinical trials for the treatment of COVID-19 pneumonia, acute pancreatitis, chronic acute liver failure, alcoholic hepatitis, and graft-versus-host disease (GVHD) (NCT02406651). Several studies have established that recombinant IL-22 has favorable pharmacological properties regarding safety, pharmacokinetics, pharmacodynamics, and tolerability [90,91].

Aside from functioning as the guardian of the intestinal barrier, IL-22 exerts antibacterial and antiviral properties by enhancing autophagy [92]. Impaired autophagy has been documented in both SCZ and IBD, while IL-22 enhances autophagy, a property of many antipsychotic drugs, including clozapine [93]. Moreover, like antipsychotics, IL-22 lowers INF-γ and protects the intestinal barrier against IBD and microbial translocation [93].

Natural human IL-22 comprises 179 amino acids and an N-terminal containing 33 amino acids. *Escherichia coli*-generated recombinant human IL-22 forms a dimer structure in solution. Recombinant IL-22 is well tolerated; it was administered by intravenous (IV) infusion in human volunteers at 2.0, 10, 30, and 45 µg/kg without severe adverse effects. A dose-dependent transient increase in serum amyloid A- and C-reactive protein was reported, as was a decrease in serum triglycerides. The compound was safe, well tolerated in animal studies, and associated with reduced inflammatory markers. 

## 13. Conclusions

The continued existence of state hospitals a century after similar institutions for communicable diseases, such as tuberculosis and leprosy, were closed, constitutes proof of the concept that outcomes in psychiatry are less robust than those of other medical disciplines.

The shortcomings of the dopamine hypothesis were highlighted by the SARS-CoV-2 virus-mediated psychosis, as well as the new SCZ findings, including the discovery of a virome in Broadman area 46, comorbidity with IBD, autoantibodies, and association with pollutants or plasticizers. 

The insufficient progress in SCZ management is probably due to the overemphasis on dopamine, while paying less attention to the measurable biomarkers of this disease, such as GMV depletion, premature senescence, or disappearance of rapid rhythms on EEGs. 

Recombinant human IL-22 represents a unique intervention strategy that may address the etiopathogenetic cause of SCZ rather than symptoms. The same may be true of AhR inhibitors, natural or synthetic compounds that act at many levels to avert the initial psychotic episode.

The advent of novel antipsychotic drugs, the first non-dopaminergic agents in over 70 years, is a step in right direction, and we expect many more to come. Furthermore, as dopamine gives away electrons, it preserves the GMV and may be a guardian against brain atrophy. Once the fear of inducing psychosis is overcome, dopaminergic drugs may start to be used in chronic psychosis along with dopamine blockers to avoid GMV loss.

The COVID-19 vaccine introduced us to liposomes which can easily cross the BBB. These vehicles can be used to carry nanograms of antipsychotic drugs directly to the neuronal networks, avoiding systemic adverse effects. This is a step that could revolutionize the treatment of mental illness; however, the study of liposome components is limited at present because of the proprietary nature of these molecules. The study of liposome components is important in order to avoid detrimental fusogens such as polyethylene glycol (PEG), previously utilized in lipophilic drug vehicles.

## Figures and Tables

**Table 1:** Some of the best-known natural and synthetic AhR antagonists with potential antipsychotic properties.

Compound	Category	References
Quercetin	Natural	[81]
Apigenin	Natural	[84]
Luteolin	Natural	[85]
HBU651	Synthetic	[86]
IK-175	Synthetic	[87]
CH-223191	Synthetic	[88]
